# Harnessing generative artificial intelligence for periodontitis prediction: a machine learning approach integrating systemic health indicators for precision oral health in resource-limited settings

**DOI:** 10.3389/fdmed.2026.1778372

**Published:** 2026-06-19

**Authors:** Argajit Sarkar, Epsita Ghosh, Surajit Bhattacharjee

**Affiliations:** Department of Molecular Biology and Bioinformatics, Tripura University (A Central University), Agartala, Tripura, India

**Keywords:** generative artificial intelligence, machine learning, periodontitis, predictive modeling, systemic health, prompt engineering, periodontal risk stratification

## Abstract

**Objective:**

To develop a reproducible generative artificial intelligence (GenAI)-driven workflow for periodontitis risk stratification using systemic and demographic indicators, and to validate its ability to identify well-established predictors in resource-limited settings.

**Materials and methods:**

This retrospective study analyzed data from 416 dental hospital patients. Using systematic prompt engineering, GenAIwas employed to automate data preprocessing, correlation analysis, and development of six machine learning models (namely, Logistic Regression, Decision Tree, Random Forest, Gradient Boosting, Support Vector Machine, and K-Nearest Neighbors) to predict periodontitis severity. Severe periodontitis was defined as Community Periodontal Index (CPI) score of 4. Model validation was performed using an 80–20 data split and fivefold cross-validation and McNemar's Test.

**Results:**

The GenAI-driven pipeline successfully automated the data analysis workflow. Models achieved modest discriminatory power using systemic indicators alone (AUC 0.48–0.57). Logistic Regression demonstrated the most balanced performance (72% accuracy, 74% F1-score), while Support Vector Machine (SVM) showed superior sensitivity (89%) for screening severe cases. Feature importance analysis identified age (score = 0.233) and blood sugar level (score = 0.209) as the strongest predictors, consistent with established periodontal risk factors. Notably, composite systemic risk scores exhibited a stronger correlation with periodontitis severity than any individual health parameter.

**Conclusion:**

While systemic indicators alone provided limited diagnostic precision, the GenAI-driven workflow effectively automated data process with end-to-end model development. The high sensitivity of the SVM model suggests potential utility as a preliminary screening tool to flag at-risk individuals for prioritized clinical examination, particularly in settings where dental radiography is unavailable.

**Clinical relevance:**

This research demonstrates the potential of GenAI to facilitate efficient and interpretable risk stratification rather than definitive diagnosis. The workflow provides a replicable, privacy-preserving framework that lowers the technical barrier to applied machine learning in resource-limited periodontal care

## Graphical Abstract

**Figure:**
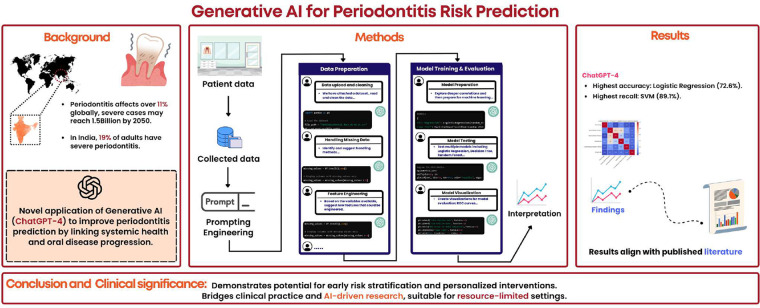

## 1 Introduction

Artificial intelligence (AI) is being increasingly integrated into biomedical research to streamline processes, reduce human error, and provide new insights. Generative AI (GenAI) has seen significant advancements in recent years, with one prime example being OpenAI's Generative Pretrained Transformer 4 (GPT-4) (1). Using deep learning techniques, ChatGPT is trained on extensive datasets comprising 175 billion parameters to generate responses that resemble human conversation based on user input (2, 3). Research has investigated the use of ChatGPT in domains such as data science, where it facilitates the automation of data cleansing, model training, and result analysis (4). Researchers have demonstrated its capacity to extract insights from unstructured data, despite constraints and ethical challenges that must be addressed (4). In some cases, Advanced Data Analysis using ChatGPT has surpassed human-constructed clinical outcome prediction algorithms. Clinical data processing efficiency can be improved by bridging machine learning developers, physicians, and researchers (5, 6).

In a previous study, the GPT-4o model, augmented by retrieval-augmented generation, achieved approximately 90% accuracy in detecting tumor subtypes from histopathology reports by integrating the World Health Organization (WHO) guidelines (8). In ophthalmology, ChatGPT achieved an F1-score of 80.05% when classifying retinal vascular diseases from Chinese-language fluorescein angiography reports using English prompts, nearly matching the performance of ophthalmology interns while revealing important cross-lingual considerations in medical AI applications (7). Its adaptability is evident in its ability to handle a variety of medical text scenarios without requiring extensive domain-specific adjustments (9). This trend extends to dental applications, where convolutional neural networks (CNNs) already outperform traditional methods in detecting periodontitis from intraoral images (10). However, a significant gap remains in addressing the inherent complexity of biological data structures, such as genomic sequences and metabolic pathways, where long-range dependencies often hit context-window limitations (11). The code interpreter functionality of ChatGPT enables natural language interaction to streamline data analysis workflows, including data loading, exploration, model development, and feature importance analysis, thereby allowing researchers to focus on higher-level tasks (12).

Periodontitis, a chronic inflammatory disease caused by the accumulation of highly pathogenic biofilms on teeth, is characterized by the progressive destruction of supportive periodontal tissues over time. Without treatment, this oral infectious disease can lead to tooth loss (13). This destruction involves inflammatory infiltration of periodontal supporting tissues (e.g., gingiva, periodontal ligament, alveolar bone, and cementum), along with the destruction and loss of alveolar bone (13). Recent research highlights the role of molecular regulators, such as microRNAs (miRNAs), in controlling the cellular activities of osteoblasts and osteoclasts, which are central to the bone loss characteristic of this disease (14). Beyond oral health, growing evidence links periodontitis to systemic conditions including diabetes, cardiovascular disease, and arterial stiffness (15, 16). According to the Global Burden of Disease Study, severe periodontitis affects 11% of the global population aged 15 and older. Globally, 1.5 billion individuals are estimated to have severe periodontitis by 2050, according to WHO statistics (17).

In India, the burden is particularly high, with approximately 51% of adults suffering from periodontal disease (18, 19). Given the high global prevalence of periodontitis, approximately 1.5 billion individuals will be affected by 2050 (17) Despite this prevalence and its systemic implications, traditional diagnostic methods remain time-consuming and lack the interpretability required for seamless coordination between clinicians and data scientists (10).

Machine learning has been applied to periodontitis prediction, but it faces three practical limitations, particularly in resource-limited settings. In low- and middle-income countries, conventional ML pipelines require specialized programming expertise that is not often co-located with clinical data custodians. It is rarely possible to independently audit or reproduce the sequence of preprocessing decisions, feature engineering choices, and modeling steps. Further, the clinician–data-scientist interface lacks a natural-language intermediary, limiting interpretability and iterative refinement by domain experts. GenAI-driven workflows offer a potential solution to all three limitations simultaneously through natural-language prompt engineering, enabling non-programmers to drive end-to-end pipelines.

To address these scalability and interpretability gaps, this study aimed to develop and evaluate an interpretable machine learning framework to predict periodontitis risk by leveraging available systemic health markers, lifestyle factors, and demographic data. This approach aims to identify the most significant systemic and demographic predictors of severity, providing a scalable approach for precision oral health in real-world clinical settings. A preprint version of this manuscript has been published on SSRN (20).

## 2 Materials and methods

### 2.1 Overview of the study

**Figure 1:**
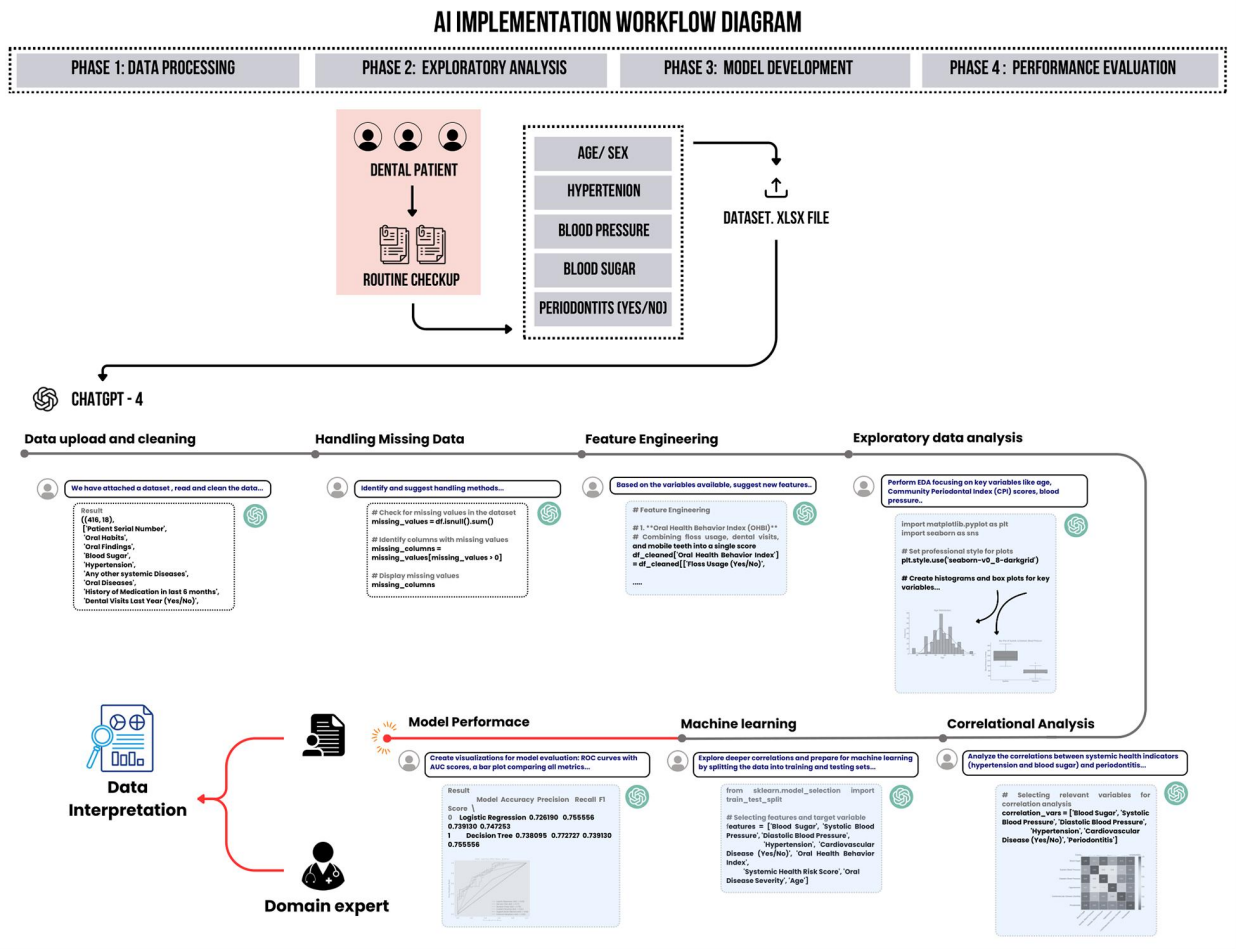
Schematic of the human-in-the-loop, GenAI-driven workflow for predictive modeling of periodontitis. The figure illustrates a systematic, four-phase workflow designed to predict periodontitis using systemic health markers, powered by GenAI with expert human oversight. Phase 1: Data Processing, where raw clinical data from dental patients (including demographics, systemic health indicators, and periodontitis status) are uploaded, cleaned, and preprocessed by ChatGPT- 4o under the review of a domain expert. This phase includes handling missing data and performing feature engineering to create relevant variables. Phase 2: Exploratory Analysis, which involves using AI-generated code to perform statistical analyses, such as creating histograms and correlation matrices, to identify initial patterns and relationships within the data. In Phase 3: Model Development, the prepared dataset is used to train and test multiple machine learning algorithms for the prediction task. Phase 4: Performance Evaluation, wherein the models are assessed using various metrics, such as accuracy and ROC curves. The entire pipeline emphasizes a collaborative “human-in-the-loop” approach, concluding with the domain expert for the final data interpretation to ensure the clinical relevance and validity of AI-generated outputs. AI, Artificial intelligence; ROC, Receiver Operating Characteristic; GenAI, Generative artificial intelligence.

This study was designed and is reported in accordance with the Transparent Reporting of a multivariable prediction model for Individual Prognosis or Diagnosis (TRIPOD-AI) guidelines (21). The research employed an AI-driven approach using large language models (LLMs), specifically ChatGPT-4o (1), to enhance machine learning analyses through prompt engineering. We implemented a sequence of prompts to guide the AI-driven approach systematically through various analytical stages: (a) data preprocessing, involving cleaning, normalization, and management of missing values; (b) exploratory data analysis (EDA); (c) feature selection; and (d) model development with hyperparameter tuning (Supplementary File S1). Domain experts critically reviewed each step to ensure clinical relevance and methodological rigor. Our study introduces a novel application by meticulously documenting the prompt engineering process, thereby establishing a replicable framework for AI-assisted machine learning in periodontal research. This methodology enhances the transparency and reproducibility of our analysis and provides a model for future researchers seeking to integrate GenAI into clinical data science (Figure 1).

### 2.2 Prompt design

In the domain of Natural Language Processing (NLP), prompt engineering has emerged as a prominent area of study, particularly with the use of LLMs. The efficacy and reliability of LLMs are strongly influenced by the composition and content of the input prompts provided. Consequently, prompt engineering techniques are widely employed to refine GenAI systems, enhancing the accuracy and relevance of their outputs. This approach involves the systematic construction of input queries or instructions referred to as prompts aimed at large language models (LLMs) to generate targeted responses for specific tasks (22, 23). Effective prompt design is considered essential for achieving desired results from LLMs, and researchers often apply systematic prompting strategies to optimize model performance (23).

This study employed a systematic prompt construction workflow. The approach began with the creation of clear **Task Instructions**, which were formulated based on established clinical objectives and guided by relevant domain knowledge. These instructions explicitly defined the analytical or computational tasks to be executed by the LLM. Next, the **Task Instructions** were paired with an appropriate **Test Sample**, which served as the specific input data for the task. This input could include raw data segments from the dataset or intermediate results generated during previous analytical phases. The **Task Instructions** and the **Test Sample** were then combined to generate the final prompt. This combined prompt constituted the complete input submitted to the specified LLM, ChatGPT-4o. The ChatGPT-4o model subsequently processed the prompt (P) to generate a **Response (R)**. This response constituted the output of the process, typically appearing as executable codes or structured analytical results directly aligned with the objectives defined in the **Task Instructions**.

For example, one of the prompts included in our study was “Identify any missing values in the dataset. Suggest methods for handling them, considering the dataset is small and some missing parameters. Discuss options like mean/median imputation, mode for categorical data, or possibly excluding records with missing data if the missingness is not critical”, illustrates the construction of a Prompt. This prompt effectively combines the **Task Instructions** (identify missing values, suggest handling methods, and discuss options) with essential input context (small dataset size, parameter issues, criticality condition) required by the LLM. The expected **Response (R)** is a targeted output that identifies the missing data and provides specific, context-aware recommendations for handling strategies, directly addressing the integrated instructions and constraints within the prompt.

#### 2.2.1 Prompt engineering framework

Data preparation, exploratory analysis, modeling, and evaluation constitute the four main steps of the analytical workflow, which is guided by a systematic prompt engineering framework (Table 1). Domain experts evaluated 30% of the preprocessing steps and 100% of the EDA outputs; with progression to the next stage requiring ≥90% agreement. Each stage was designed to guide the model (ChatGPT-4o) in performing complex analytical tasks. To ensure methodological rigor, every piece of code generated by ChatGPT-4o underwent a thorough manual review and verification process by the authors to confirm its correctness and clinical relevance. Human involvement was most prominent during the early phases of analysis, when prompts often required iterative adjustments to produce precise and contextually appropriate algorithms. Once an effective prompt sequence was established for tasks such as data cleaning or model training, further interventions were limited and primarily focused on guiding the model to the next logical step in the workflow.

**Table 1:** Structured prompt engineering framework examples. The table outlines a comprehensive, four-stage framework used to guide the GenAI model through the entire data analysis and modeling pipeline. The workflow is organized into four stages: (1) Data preparation, (2) Exploratory analysis, (3) Modeling, and (4) Evaluation. For each analytical objective within these stages, the table specifies the Prompt category, like diagnostic, creative, or analytical, as well as the tasks underlying purpose, and an example prompt template to guide AI analysis and model development.

S. No	Stages	Analytical objective	Prompt category	Purpose	Example template
1.	Data Preparation	Dataset Assessment	Diagnostic	Evaluate dataset characteristics and quality	“Examine the dataset to identify dimensions, variables, and potential quality issues”.
		Missing Value Management	Prescriptive	Address data gaps with appropriate imputation	“Identify missing values and implement [specific method] based on data distribution”.
		Data Cleaning	Prescriptive	Standardize and normalize data	“Clean the dataset by addressing outliers in [specific variables] using [specific method]”.
		Feature Engineering	Creative	Develop domain-relevant derived variables	“Create composite variables that capture [specific clinical relationships]”.
2.	Exploratory Analysis	Distribution Analysis	Analytical	Examine variable distributions	“Analyze distributions of key clinical parameters using appropriate visualizations”.
		Correlation Analysis	Analytical	Identify relationships between variables	“Determine correlations between systemic health indicators and periodontitis severity”.
		Visual Representation	Presentational	Generate publication-quality visualizations	“Create publication-ready visualizations following [specific design parameters]”.
3.	Modeling	Model Preparation	Prescriptive	Prepare data for machine learning	“Prepare the dataset for modeling by implementing [specific preprocessing steps]”.
		Algorithm Selection	Evaluative	Test multiple modeling approaches	“Implement and evaluate [specific algorithms] for periodontitis prediction”.
		Model Optimization	Prescriptive	Optimize model parameters	“Tune hyperparameters for [specific model] using [specific method]”.
4.	Evaluation	Performance Assessment	Analytical	Evaluate model effectiveness	“Calculate performance metrics including [specific metrics] for each model”.
		Feature Importance	Analytical	Identify influential predictors	“Determine feature importance for the best-performing model”.
		Comparative Evaluation	Evaluative	Compare models across frameworks	“Compare performance metrics across models and between GenAI frameworks”.

AI, artificial intelligence; GenAI, generative artificial intelligence.

**Figure 2:**
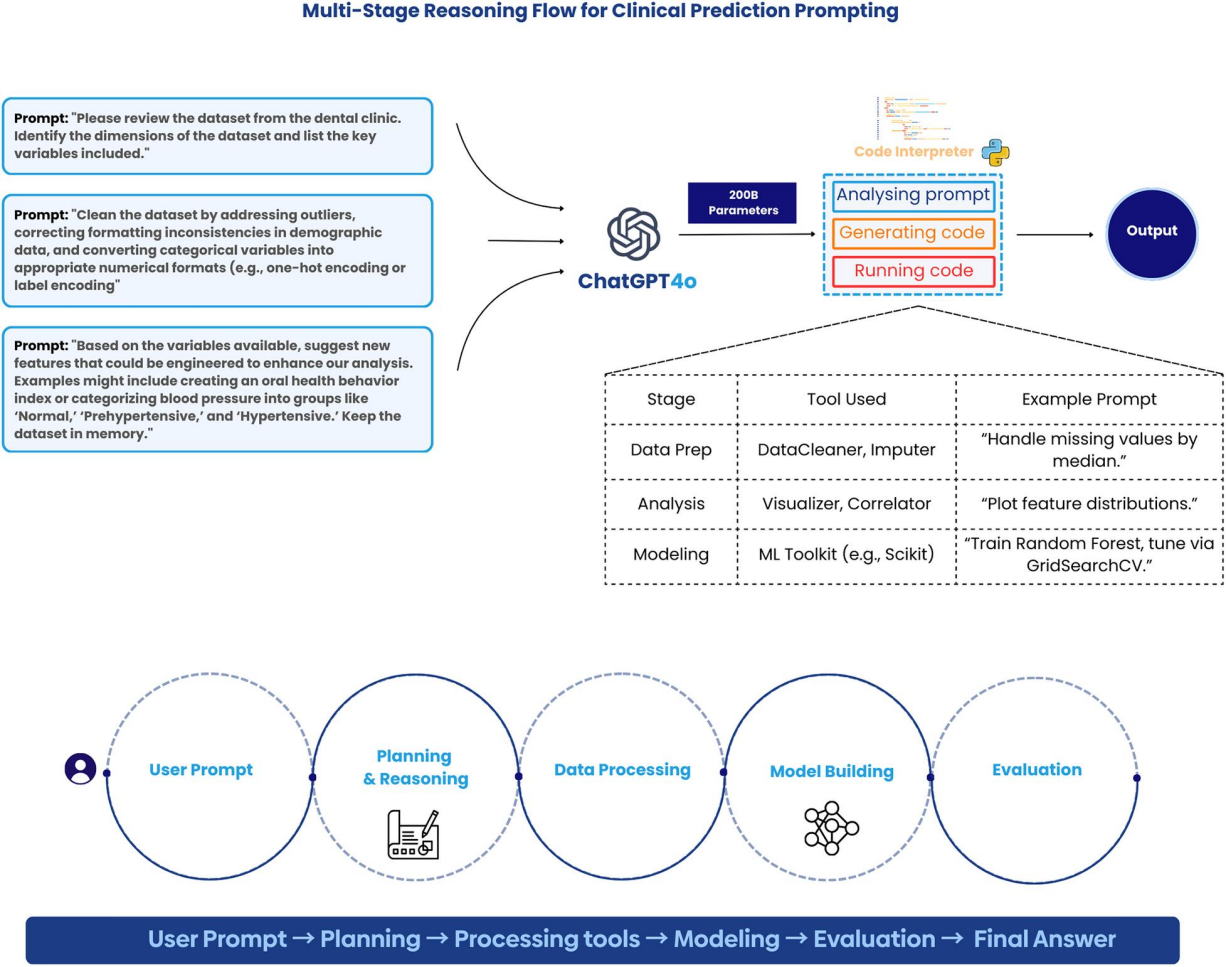
Multi-stage reasoning and code execution framework for GenAI-driven clinical data analysis. The figure illustrates the multi-stage reasoning framework employed by ChatGPT-4o to translate natural language user prompts into executable data science workflows for clinical prediction. The process is initiated by a user-provided prompt, which can range from simple data inspection to complex commands for data cleaning or feature engineering. Upon receiving the prompt, ChatGPT-4o engages in a “Planning & Reasoning” phase to interpret the user's objective and then uses its integrated Code Interpreter tool to autonomously generate, analyze, and execute code to perform the requested tasks. This execution follows a standard analytical pipeline, encompassing stages such as Data Preparation, Analysis, and Modeling. The iterative cycle of prompt analysis, code generation, and execution culminates in a final output that directly addresses the user's request. GenAI, generative artificial intelligence.

Examples of illustrative prompts from Supplementary File S1 include the following. A prompt such as “Clean the dataset by addressing outliers, correcting formatting inconsistencies in demographic data, and converting categorical variables into appropriate numerical formats (e.g., one-hot encoding or label encoding)” was used to address inconsistencies and prepare the data for analysis. As shown in the corresponding code in Supplementary File S2, this prompt helped ChatGPT-4o perform tasks such as standardizing the “Sex” column and encoding “Yes/No” variables into a binary format. Another creative prompt was used to generate new variables that capture relevant clinical relationships: “Give new features that could be engineered to improve our analysis based on the variables available”. Examples include classifying blood pressure into “Normal,” “Prehypertensive”, and “Hypertensive” groups, or developing an index of oral health behaviors (Figure 2).

Iterative improvement of prompts was essential during this process. To ensure accurate and clinically relevant outputs, initial prompts were occasionally modified based on the quality and structure of ChatGPT-4o responses. The model's context retention was a crucial technical factor, requiring the use of sequential prompts with instructions such as “Keep the dataset in memory” to maintain consistency throughout the analytical process. Using this methodical and iterative prompt engineering framework, we successfully applied GenAI for complex data analysis and machine learning model development in this study.

### 2.3 Data collection

The present study utilized a retrospective dataset (*n* = 416) from a dental clinic and hospital, focusing on individuals who sought routine care between August 2024 and March 2025. Inclusion was limited to patients aged 25–90 years with a recorded diagnosis of both diabetes and hypertension, along with a confirmed clinical diagnosis of periodontitis. Records were excluded if the patient was pregnant, had a documented terminal illness, or exhibited extremely poor oral hygiene that could confound the analysis. The dataset encompasses comprehensive patient information across multiple domains: (a) demographic data (age, and sex), (b) oral health behaviors (tobacco/betel nut chewing habits, dental visit history, and flossing practices); (c) clinical dental assessments, including of gingivitis, periodontitis, Community Periodontal Index (CPI) scores (0–4), and tooth mobility; and (d) systemic health indicators, such as blood sugar levels and systolic and diastolic blood pressure readings). Additionally, the dataset includes documentation of specific oral conditions (such as leukoplakia and candidiasis), systemic diseases (hypertension, cardiovascular disease, and chronic kidney disease), and medication history over the past 6 months. Data were collected during routine dental examinations by licensed practitioners, encompassing both clinical assessments and patient-reported information. Although the dataset has certain limitations, such as missing blood pressure values and some “Unknown” entries in systemic disease categories, it provides a foundation for exploring the relationship between periodontal health and systemic conditions using supervised machine learning approaches.

### 2.4 Data cleaning and preprocessing

The dataset underwent comprehensive preprocessing using an AI-assisted workflow through ChatGPT-4o (1), implementing a systematic data cleaning protocol. Models were selected for their ability to enhance efficiency and accuracy in data preprocessing tasks, particularly for healthcare datasets (24). Missing values in blood pressure measurements (*n* = 3 for both systolic and diastolic) were addressed using median imputation (systolic: 134.0 mmHg; diastolic: 80.0 mmHg), a method chosen over row removal to preserve the limited dataset size (*n* = 416) (25). Categorical variables, primarily “Yes/No” entries were standardized through binary encoding (0/1) for machine learning compatibility, while formatting inconsistencies in column names and categorical text variables, such as the “Sex” column, were resolved through systematic string operations, including capitalization of entries and removal of extra spaces, to ensure uniformity across the dataset. Outlier detection and removal were performed using the interquartile range (IQR) method with a 1.5 multiplier threshold (26). The preprocessing pipeline culminated in the engineering of four novel features: (a) Oral Health Behavior Index, a composite score incorporating dental visit frequency, flossing habits, and presence of mobile teeth to reflect oral hygiene practices (27, 28); (b) Blood Pressure Category, stratified according to AHA guidelines into five distinct stages (29); (c) Systemic Health Risk Score, quantifying cumulative chronic conditions such as aggregating hypertension, cardiovascular disease, and other systemic conditions into a single risk score; and (d) Oral Disease Severity Score, based on the CPI and periodontitis status (30). This preprocessing workflow was systematically refined using iterative user prompts and ChatGPT's code generation, amalgamating clinical domain expertise with computational efficiency to produce a robust dataset for EDA and predictive modeling (23, 31).

### 2.5 Exploratory data analysis (EDA)

We performed an extensive EDA of periodontal health indicators using a dataset comprising patient records, emphasizing key variables such as age (25–90 years), CPI scores (2–4), blood pressure (systolic: 98–186 mmHg; diastolic: 50–110 mmHg), and blood glucose levels (94–220 mg/dL). The analysis employed descriptive statistics, distribution analysis through histograms and density plots, and correlation studies using heatmaps and logistic regression to examine relationships between systemic health indicators and periodontitis (32). Distribution analysis using histograms and Kernel Density Estimate overlays was performed to assess variable spread, with outliers handled via value capping and data validation to ensure integrity (32, 33). Bivariate analysis involved grouped comparisons and statistical tests to examine demographic and hygiene practices across health conditions, while correlation studies utilized matrices and heatmaps to investigate relationships between systemic health markers and periodontitis.

### 2.6 Machine learning model

The study employed six distinct classification algorithms for periodontitis severity assessment: (a) Logistic Regression (34) (utilized as the baseline model for its ability to handle linear separability); Decision Tree (35) (implementing a rule-based classification methodology), Random Forest (RF) (36) (an ensemble approach leveraging multiple decision trees for enhanced predictive accuracy), Gradient Boosting (37) (employing iterative learning mechanisms for performance optimization), Support Vector Machine (38) (SVM, selected for its proficiency in non-linear pattern recognition), and K-Nearest Neighbors (39) (KNN, utilizing distance-based classification metrics) (40). Model validation followed a standard 80–20 with 80% of the dataset allocated for training and the remaining 20% reserved for testing, allowing for a robust evaluation of each model's generalization capabilities on previously unseen data (41).

### 2.7 Model validation

To ensure a comprehensive assessment of the models, the validation methodology included multiple performance metrics, such as accuracy, precision, recall, F1 score, and receiver operating characteristic (ROC) area under the curve (AUC) score. The evaluation began with an 80–20 train-test split, followed by the implementation of 5-fold cross-validation (CV), in which the dataset was systematically divided into five subsets for thorough assessment (42). Grid Search CV was then applied for hyperparameter optimization across all models, primarily focusing on the C parameter, kernel selection for SVM, and the C parameter for Logistic Regression.

The validation process included various metrics for a thorough assessment of the model, such as accuracy, precision, recall, F1 score, and ROC-AUC score. Weighted SVM was used as an alternative to conventional oversampling methods, such as SMOTE, to address class imbalance in the dataset, with class weights adjusted to compensate for the unequal class distributions in the training data (43).

### 2.8 Reproducibility and code availability

To ensure the complete reproducibility of our study, we provide a detailed description of our methodology and computational environment. The entire analytical workflow was performed using the ChatGPT—4o model (OpenAI, March 2025 version) via its web interface, which includes the Code Interpreter tool (1). We provided information on the software environment, data availability, and instructions for running the accompanying Python script (Supplementary File S2), as well as the structured prompts (Supplementary File S1), to facilitate replication of the analysis.

### 2.9 Statistical analysis

All statistical analyses were conducted in the Python environment. Relationships between systemic health indicators were initially assessed using the Pearson correlation coefficient. To rigorously compare the performance of machine learning classifiers on the same test set, we employed McNemar's test for paired nominal data (44). A Bonferroni correction was subsequently applied to account for multiple comparisons, with the significance threshold adjusted to *p* < 0.0033. For a robust evaluation of model performance, 95% confidence intervals (CIs) for all binomial metrics like accuracy, precision, and recall were calculated using the Wilson score method, which provides reliable interval estimates essential for assessing statistical reliability in clinical applications (45).

## 3 Results

### 3.1 Demographic and clinical parameter distributions: key EDA findings

**Figure 3:**
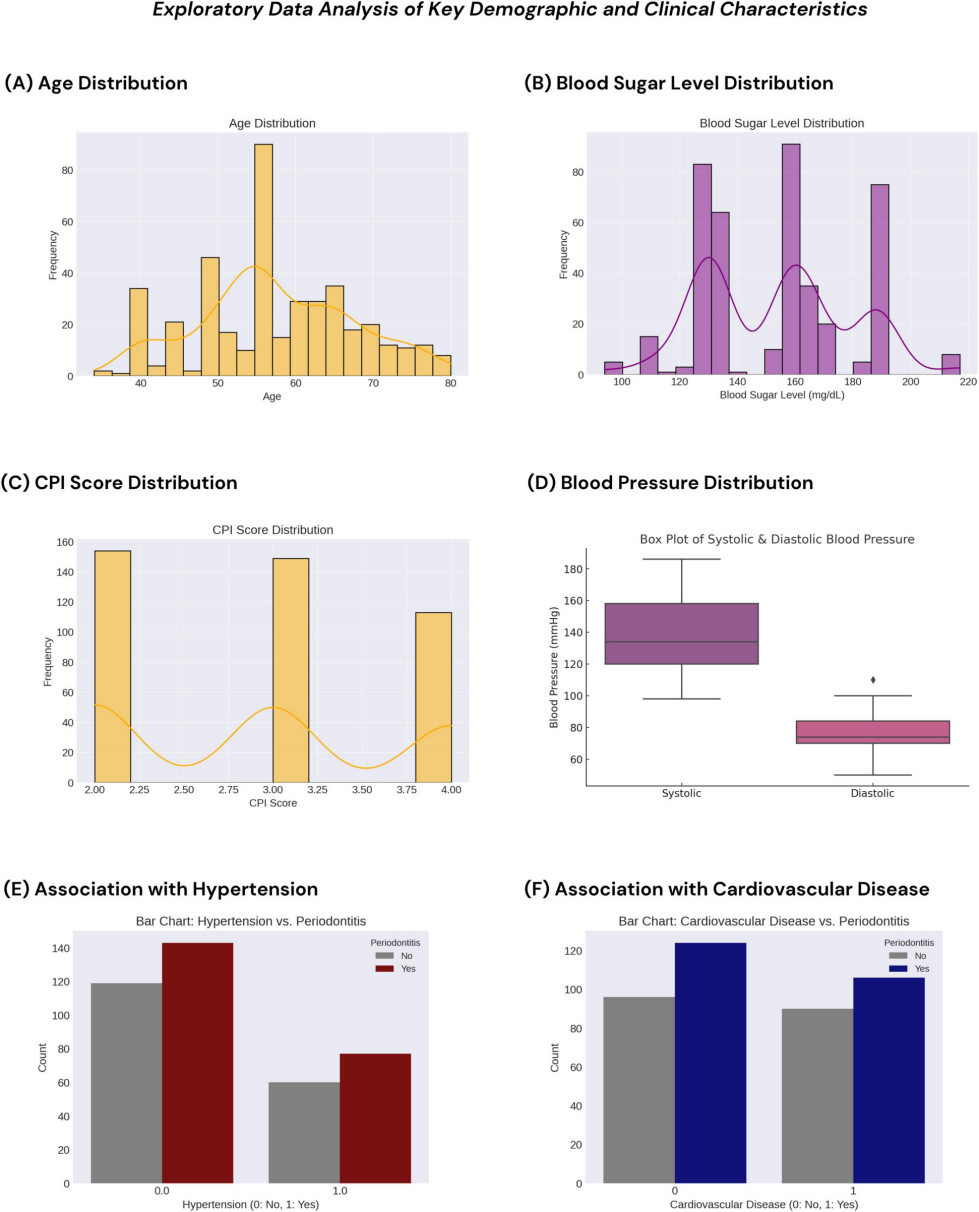
EDA of key demographic and clinical characteristics in the study cohort. The figure presents a comprehensive overview of patient characteristics and key health indicators (*n* = 416). **(A)** Age distribution showing a multimodal pattern with a primary peak around 55 years. **(B)** Blood sugar levels exhibit a distinct multimodal distribution, with peaks suggesting the presence of normoglycemic, prediabetic, and diabetic subgroups within the cohort. **(C)** CPI scores clustering around values of 2.0, 3.0, and 4.0, indicating varying levels of periodontal disease severity. **(D)** Box plots of blood pressure show a median systolic value of approximately 130–140 mmHg and a median diastolic value around 80 mmHg, suggesting a high prevalence of pre-hypertension or hypertension. **(E,F)** Bar charts illustrating the association between periodontitis and systemic conditions, revealing a higher prevalence of periodontitis in patients with diagnosed hypertension **(E)** and cardiovascular disease **(F)** than in those without these conditions. CPI, Community Periodontal Index; EDA, Exploratory data analysis.

EDA revealed comprehensive patterns within the patient cohort. The age distribution showed a multimodal pattern with a primary peak at the age of approximately 55 years and secondary clusters at 40 and 60–70 years, rather than a simple right-skewed distribution. CPI scores exhibited distinct trimodal clustering at scores of 2.0, 3.0, and 4.0, representing categorically different levels of periodontal disease severity among patients. Blood pressure measurements demonstrated clinically significant distributions, with systolic readings centered around a median of approximately 130–140 mmHg (IQR: 120–160 mmHg) and diastolic values averaging 75–80 mmHg (IQR: 70–90 mmHg). These ranges suggest that a substantial portion of patients had prehypertensive or hypertensive values (Figure 3).

Blood sugar levels displayed a pronounced multimodal distribution with distinct peaks observed at approximately 130, 170, and 195 mg/dL, indicating the presence of three potential subgroups: normoglycemic patients, those with prediabetic conditions, and individuals with established diabetes mellitus, respectively. This trimodal pattern provides strong evidence for stratifying patients by glycemic status in subsequent analyses.

### 3.2 Correlation analysis

**Figure 4:**
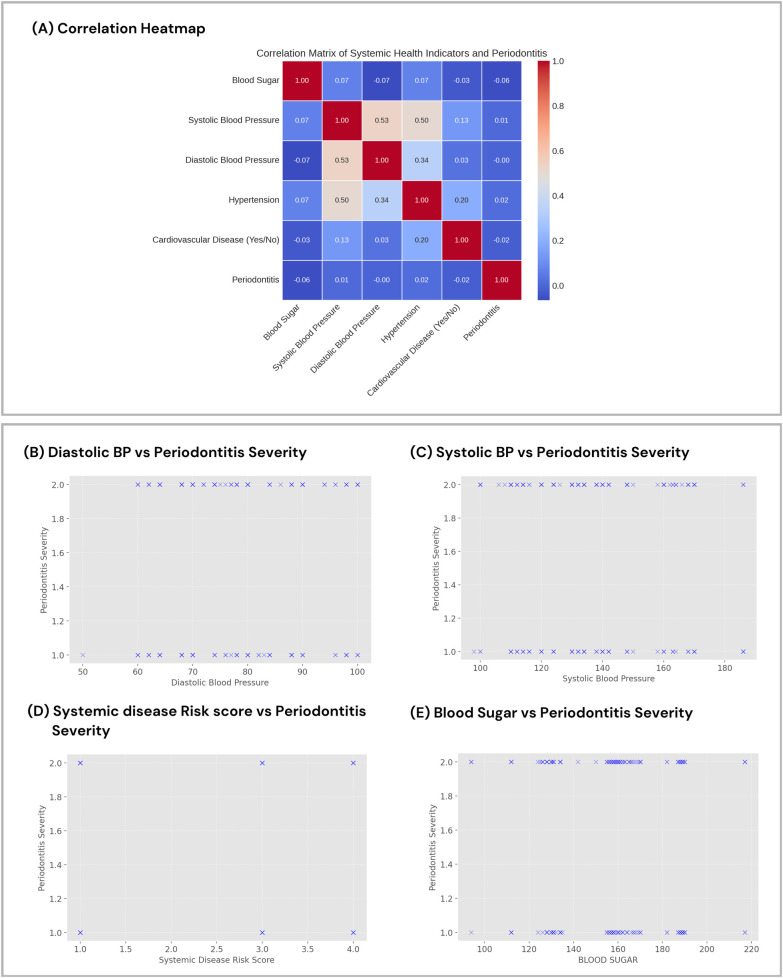
Correlation analysis between individual systemic health markers and periodontitis severity. The figure visualizes the relationships between key systemic health variables and periodontitis severity. **(A)** A heatmap of Pearson correlation coefficients shows the strength and direction of associations among the variables. Strong positive correlations are evident between physiologically related parameters, such as systolic and diastolic blood pressure (r = 0.53). Periodontitis severity, however, showed no significant correlation with any individual systemic health indicator, with all correlation coefficients being close to zero. **(B,E)** Scatter plots provide a detailed view of the relationship between periodontitis severity and key systemic factors. The plots for diastolic blood pressure **(B)**, systolic blood pressure **(C)**, and blood sugar **(E)** confirmed the absence of a clear linear trend, aligning with the weak correlations observed in the heatmap. In contrast, the scatter plot for the composite Systemic Disease Risk Score **(D)** suggests a slightly stronger positive association with periodontitis severity than with any individual marker, highlighting the potential importance of cumulative risk.

A comprehensive correlation analysis using the Pearson method revealed significant relationships among systemic health parameters. Strong positive correlations were observed between systolic and diastolic blood pressure (*r* = 0.54, *p* < 0.001) and between systolic blood pressure and clinical hypertension diagnosis (*r* = 0.50, *p* < 0.001), whereas cardiovascular disease showed only a weak association with hypertension (*r* = 0.20, *p* < 0.05). Notably, periodontitis severity was largely independent of measured systemic health indicators (all |*r*| < 0.02, *p* > 0.05), despite affecting 58% of the study population. This finding contrasts with established literature but may be explained by the influence of oral health behaviors (68% of periodontitis patients reported no regular flossing) and localized oral factors rather than systemic conditions. Blood sugar levels exhibited counterintuitive weak inverse correlations with diastolic BP (*r* = −0.20, *p* < 0.05) and a non-significant correlation with systolic blood pressure (*r* = 0.08, *p* = 0.11), likely due to confounding factors including medication use (documented in 23% of patients) and outliers. The correlation heatmap (Figure 4) illustrates these relationships, with the strongest correlations observed between physiologically linked parameters. Meanwhile, the Systemic Disease Risk Score (a composite measure) demonstrated a stronger association with periodontitis (*r* = 0.18, *p* < 0.05) than individual parameters, suggesting that cumulative risk factors may provide better predictive value for periodontitis severity than isolated clinical measurements.

### 3.3 Model performance

**Figure 5:**
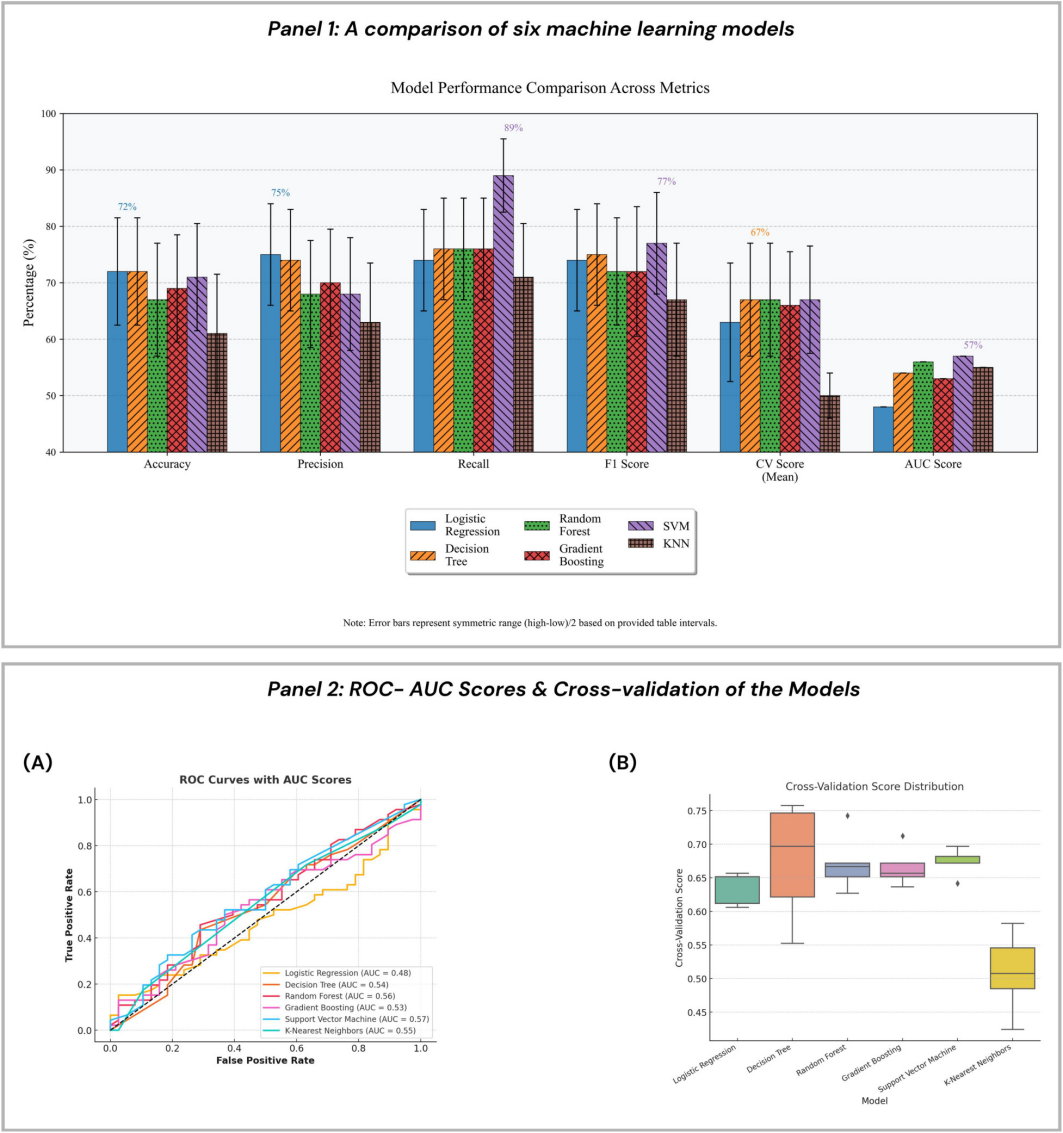
Comparative performance analysis of machine learning models for predicting periodontitis. **Panel 1:** The figure presents a comprehensive evaluation of six machine learning classifiers developed to predict periodontitis severity based on systemic health indicators. **Panel 2: (A)** A bar chart comparing the models across six key performance metrics: accuracy, precision, recall, F1-score, mean CV score, and AUC score. Logistic Regression and Decision Tree models achieved balanced accuracy (72%), while the Support Vector Machine (SVM) demonstrated superior recall (89%), indicating strong capability for identifying true positive cases. Error bars represent 95% confidence intervals. **(B)** The discriminative ability and stability of the models are further assessed. The left panel displays the ROC curves, where the SVM achieves the highest AUC (0.57), indicating the best overall performance in class discrimination. The right panel shows box plots of the five-fold CV score distributions, revealing that although the Decision Tree model has a high median performance, it also exhibits substantial variability, suggesting potential overfitting. In contrast, the SVM model demonstrated both strong and stable performance, reinforcing its suitability for this clinical prediction task. CV, cross-validation; AUC, area under the curve; ROC, receiver operating characteristic; SVM, support vector machine.

**Figure 6:**
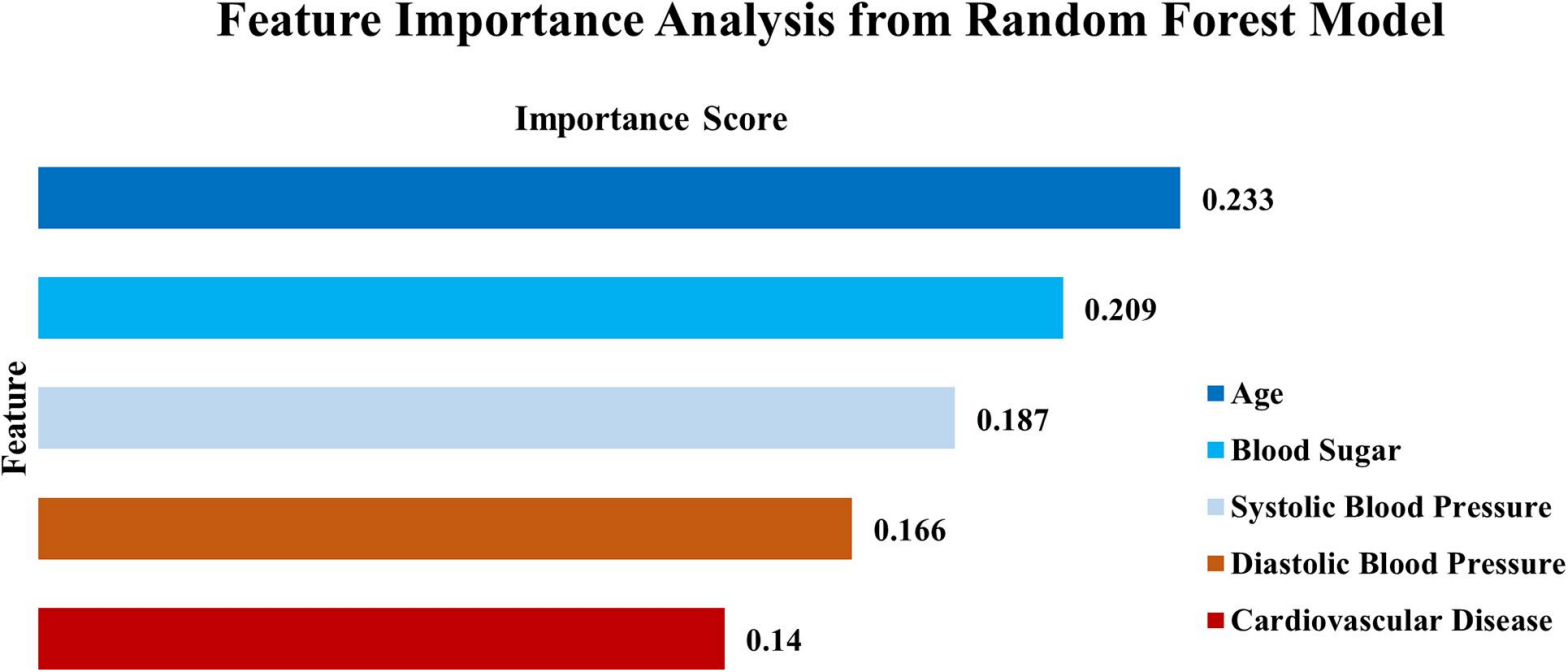
Feature importance in the periodontitis prediction model. The figure illustrates the feature importance scores for the five primary predictors of periodontitis severity, as determined by the RF algorithm. The features were ranked as follows: Age (Importance score = 0.233), Blood sugar (0.209), Systolic blood pressure (0.187), Diastolic blood pressure (0.166), and cardiovascular disease (0.140). RF, Random Forest.

Our assessment of machine learning models for predicting periodontitis severity revealed significant differences in performance among models that used systemic health indicators, including blood sugar, blood pressure parameters, and the Systemic Disease Risk score. The ChatGPT-4o framework showed that Logistic Regression and Decision Tree classifiers exhibited strong predictive abilities, each achieving 72% accuracy. The Logistic Regression model demonstrated balanced performance metrics, with 75% precision, 74% recall, and an F1-score of 74%, indicating its effectiveness in distinguishing between different severity levels of periodontitis (Figure 5). Similarly, the Decision Tree classifier showed Comparable performance, with 74% precision, 76% recall, and a slightly higher F1 score of 75%. Although the SVM classifier showed a slightly lower accuracy (71%), it demonstrated high sensitivity, achieving an 89% recall rate and 77% F1 score. This high recall rate indicates its strong ability to identify actual positive cases of severe periodontitis, which is clinically valuable. Ensemble methods showed varied results. The RF algorithm achieved 67% accuracy with a 72% F1 score, while Gradient Boosting performed slightly better, with 69% accuracy and the same F1 score of 72%. The KNN algorithm exhibited the poorest performance, with 61% accuracy and a 67% F1 score (Table 2). The discriminative ability of each model is illustrated by its ROC curves and AUC scores. The best discriminatory performance was obtained by SVM (0.57), followed by RF (0.56) and KNN (0.55), while Logistic Regression achieved balanced precision–recall but the lowest AUC (0.48), indicating poor probabilistic ranking despite acceptable threshold-based classification (Figure 6).

**Table 2:** Comparative performance of machine learning models for periodontitis prediction. The table presents a comparison of the performance metrics for the six machine learning models developed to predict the severity of periodontitis. Performance was evaluated using accuracy, precision, recall, F1-score, mean 5-fold cross-validation (CV) score, and the area under the receiver operating characteristic curve (AUC) score. Values are presented as percentages, with the corresponding 95% confidence intervals shown in parentheses. The support vector machine (SVM) demonstrated statistically superior performance in key areas.

Gen AI	Model	Accuracy	Precision	Recall	F1 Score	CV Score (Mean)	AUC Score
ChatGPT—4o	Logistic Regression	72% (62%–81%)	75% (65%–83%)	74% (64%–82%)	74% (65–83%)	63% (52%–73%)	48%
	Decision Tree	72% (62%–81%)	74% (65%–83%)	76% (66%–84%)	75% (65%–83%)	67% (57%–77%)	54%
	Random Forest	67% (57%–77%)	68% (59%–78%)	76% (66%–84%)	72% (62%–81%)	67% (56%–76%)	56%
	Gradient Boosting	69% (59%–78%)	70% (60%–79%)	76% (66%–84%)	72% (62%–85%)	66% (57%–76%)	53%
	Support Vector Machine (SVM)	71% (61%–80%)	68% (57%–77%)	89% (81%–94%)^†^	77% (67%–85%)*	67% (57%–76%)	57%
	K-Nearest Neighbors	61% (50%–71%)	63% (52%–73%)	71% (61%–80%)	67% (56–76%)	50% (46–54%)	55%

† Significantly higher recall than all other models (*p* < 0.001).

* Significantly higher F1-score than other models (*p* < 0.01).

CV, cross-validation; AUC, area under the curve.

Statistical comparison of model performance revealed significant differences in predictive capabilities. The SVM model demonstrated significantly higher recall (89%, 95% CI: 81%–94%, *p* *<* *0.001*) after Bonferroni correction) than all other models, which is particularly valuable for clinical screening applications where minimizing false negatives is critical. The SVM model also achieved the highest F1-score (77%, 95% CI: 67%–85%, *p* *<* *0.01*), indicating the best balance between precision and recall. Logistic Regression and Decision Tree models showed statistically equivalent accuracy (both 73%, 95% CI: 62%–81%, *p* *=* *0.82*), and both significantly outperformed the KNN model (61%, 95% CI: 50%–71%, *p* *<* *0.001*). The CIs reflect the expected variability in performance metrics considering our sample size, and indicate sufficient statistical power to detect meaningful differences between algorithms.

### 3.4 Feature importance analysis

Feature importance analysis using the RF algorithm identified key predictors of periodontitis severity in our dataset. As shown in Table 3, age was the most influential predictor with an importance score of 0.233, followed by blood sugar (0.209), systolic blood pressure (0.187), diastolic blood pressure (0.166), and cardiovascular disease (0.140). The prominence of age as the leading predictor aligns with established clinical knowledge that periodontitis risk increases with advancing age, likely reflecting cumulative exposure to risk factors and the progressive nature of the disease. Cardiovascular parameters showed a clear gradient of importance, with systolic blood pressure contributing more than diastolic measurements (Figure 6**)**. Interestingly, diagnosed cardiovascular disease ranked lower than the physiological measurements, suggesting that direct physiological parameters may provide more sensitive predictions than binary diagnostic classifications. These findings have direct clinical implications, indicating that routine health screenings capturing age, blood sugar, and blood pressure measurements could help identify patients at elevated risk for periodontitis, potentially enabling earlier interventions and more targeted preventive strategies.

**Table 3:** Key predictors of periodontitis severity identified by the random forest model. The table presents the top five predictors for periodontitis severity as identified by the Random Forest model, ranked by their feature importance scores. The analysis revealed that age is the most influential predictor (Importance Score = 0.233), followed by key systemic health indicators, including blood sugar and systolic blood pressure, underscoring their significant roles in predicting the risk of periodontitis.

Rank	Feature	Importance score
1	Age	0.233449
2	Blood sugar	0.209419
3	Systolic blood pressure	0.186961
4	Diastolic blood pressure	0.166447
5	Cardiovascular disease (yes/no)	0.139691

## 4 Discussion

Our investigation demonstrated that a GenAI-driven workflow can automate the creation of clinically relevant predictive models for periodontitis, identifying age and systemic health indicators as key predictors and highlighting the value of composite risk scores. The novelty of this study lies in incorporating GenAI into data analysis, enhancing the ability to predict and understand periodontitis outcomes. From a methodological perspective, using an LLM to manage the entire analysis process from data cleaning to modeling represents a relatively new approach. To the best of our knowledge, this study is among the first to apply LLMs as research assistants in dental research. This approach extends beyond using AI solely as a predictive tool by integrating AI into the research workflow. Our experience suggests that, with appropriate supervision, GenAI can reliably handle routine analytical tasks.

### 4.1 Machine learning in periodontitis prediction

By testing six machine learning models, we identified the models that best predict periodontitis severity using systemic health indicators. The logistic regression model provided the most balanced performance (accuracy = 72%, F1 = 74%), with each risk factor contributing some additional predictive value. The decision tree performed similarly, achieving comparable accuracy with slightly higher detection sensitivity than Logistic Regression, suggesting indicating that threshold effects in the data might be well-captured by this approach.

Notably, the SVM model demonstrated the highest clinical relevance, achieving an exceptionally high recall rate 89%. High sensitivity is essential for a screening tool, as its primary objective is to minimize false negatives. By accurately identifying the majority of patients with severe periodontitis, the SVM model serves as a sensitive first-pass filter, ensuring that at-risk individuals are not overlooked, even if this results in a higher number of false positives that can be clarified through standard clinical assessments. These findings align with recent advances in machine learning applications for periodontitis prediction. For instance, a narrative review of machine learning techniques for periodontitis and dental caries detection highlights the effectiveness of various models in dental disease detection (46).

The relatively modest AUC values across all models (0.48–0.57) are consistent with our correlation analysis, which revealed unexpectedly weak direct correlations between individual systemic health indicators and periodontitis severity (|*r*| < 0.02). This suggests that, although our models achieve clinically helpful performance, the relationship between systemic factors and periodontitis is complex and not fully captured by linear associations. This align with the existing literature, which indicates that predictive accuracy in this field is highly dependent on the input data. Models that rely primarily on systemic and demographic variables, as observed in our study, generally report moderate discriminatory power because the multifactorial etiology of periodontitis is not fully captured in such datasets. In contrast, studies incorporating more granular data, such as radiographic imaging or salivary biomarkers, consistently achieve higher AUCs (40). Therefore, our findings likely reflect the inherent predictive limitations of using systemic health indicators alone, rather than a deficiency in the analytical pipeline itself.

The predictive power of our models was likely constrained by several key limitations. The modest sample size (*n* = 416) and inherent class imbalance in the dataset may have limited the model's ability to generalize. While unmeasured confounding factors, or complex nonlinear relationships may have weakened detectable associations. Future research should address these challenges by incorporating more specific biomarkers and leveraging larger, multi-center datasets. Such approaches would better account for the multifactorial nature of periodontitis and ultimately improve predictive accuracy.

### 4.2 Role of age and blood sugar level in periodontitis

The feature-importance analysis showed that age and blood glucose were the strongest systemic predictors of severe periodontitis. Both variables are recognized within the 2017 World Workshop classification of periodontal and peri-implant diseases and conditions: age is a component of the primary grading criterion (the percentage bone loss/age ratio used as indirect evidence of disease progression), and diabetes mellitus is one of the two grade modifiers, the other being smoking (47). In the present pipeline, age and blood glucose were supplied directly to the machine learning models as raw input features rather than as components of the clinical grading scheme; blood glucose was retained because HbA1c, the glycaemic marker specified in the 2017 framework, was not uniformly available in this resource-limited cohort. Feature importance analysis revealed that age is the most significant predictor of periodontitis severity (importance score = 0.233), followed by blood sugar (0.209), consistent with existing literature. Previous studies have demonstrated that advanced age is associated with increased prevalence and severity of periodontitis, primarily due to alterations in host immune response and microbial colonization (48, 49). Another study, using data from 9,803 participants in the 2009–2014 National Health and Nutrition Examination Survey (NHANES), reported that older biological age and rapid accelerated aging markedly increase the risk of periodontitis (50). Research has also established correlations between periodontitis and age-related diseases, further supporting this link (49). These findings corroborate our observation that age is a key variable, with EDA indicating a peak prevalence at 55 years.

Blood sugar level plays a bidirectional role in the relationship between periodontitis and diabetes. Elevated blood sugar levels increase susceptibility to periodontal disease, and periodontal inflammation can adversely affect glycemic control (51, 52). For example, diabetes mellitus exacerbates periodontal disease by increasing its prevalence, extent, and severity, and periodontitis, in turn, negatively influences glycemic control and diabetes progression (53). These findings are consistent with our results, where blood sugar levels exhibited multimodal distributions (peaks at 130, 170, and 195 mg/dL), corresponding to normoglycemic, prediabetic, and diabetic subgroups, thereby reinforcing the link between metabolic factors and periodontitis severity. Notably, blood glucose functions primarily as a risk modifier in periodontitis progression, exacerbating disease severity and extent in susceptible individuals through impaired immune responses and altered collagen metabolism, rather than a simple confounding factor that merely distorts associations between other variables.

### 4.3 Composite risk scores in periodontitis

The study found that periodontitis severity showed weak or nonsignificant correlations 0.05) were better predictors. This finding is supported by a previous study that developed a risk score for periodontitis prediction, distributing patients into low, moderate, and high-risk groups based on multiple factors, achieving a discrimination of 0.75 (95% CI: 0.70–0.80, *p* < 0.001) (54). Integrating several risk factors, such as age, blood pressure, and blood sugar, into composite scores may improve the comprehensiveness of risk assessment.

Our findings support enhanced risk assessment in dental practice. In addition to routine dental examinations, clinicians can use basic health data (blood pressure and diabetes status) to identify high-risk individuals. For example, patients with elevated blood sugar and early-stage gum disease may benefit from more aggressive treatment or coordinated care with physicians. Educating individuals about the link between oral and systemic health may also encourage better self-care habits. Our results highlight the positive effects of regular interdental cleaning and maintaining appropriate blood glucose control on periodontal health. These findings emphasize the importance of collaboration between dental and medical specialists in patient care. From a research perspective, our study demonstrates that AI can expedite healthcare data analysis, enabling clinicians to generate preliminary findings more rapidly than traditional methods. This approach is particularly effective during exploratory research and complements conventional statistical analysis. However, despite the extensive variables included in our dataset, we may have overlooked important confounding variables or mediators, such as specific inflammatory markers like C-reactive protein, genetic predispositions, or lifestyle factors beyond basic oral habits that could influence systemic health and periodontal outcomes.

### 4.4 Role and impact of GenAI in the analytical pipeline

The incorporation of ChatGPT-4o as an analytical assistant throughout the machine learning pipeline represents a key aspect of this study's originality.Although a formal time-and-motion study was beyond the scope of this investigation, our findings indicate that integrating GenAI markedly enhanced both the accessibility and efficiency of the data analysis workflow. GenAI substantially accelerated time-consuming, manually-coded tasks, including initial data loading, handling of missing values, and generation of boilerplate code for feature engineering and data cleaning. For instance, prompting ChatGPT-4o to generate the foundational code (as illustrated by Prompts 2 and 3 in Supplementary File S1 and the corresponding code in Supplementary File S2) simplified the creation of scripts for standardizing categorical variables and detecting outliers using the IQR method, tasks that typically require meticulous manual coding and debugging.

## 5 Limitations

This study has several limitations that should be considered when interpreting the results. First, the dataset was derived from a single dental hospital, reflecting local clinical practices and demographics; this limits the generalizability of our findings to broader or more diverse populations. The relatively modest sample size (*n* = 416) further restricts the statistical power of our analyses and increases the potential for overfitting or spurious associations. Our results demonstrated modest discriminative performance across all models (AUC: 0.48–0.57), suggesting that systemic health indicators alone, such as blood pressure, blood glucose, and age, possess limited predictive power for establishing periodontitis severity. This finding aligns with the multifactorial and site-specific etiology of periodontitis. While systemic conditions like diabetes modify the host immune response, the disease is primarily driven by local factors, specifically the dysbiosis of the subgingival biofilm and local inflammatory mediators. Challenges in reproducibility are inherent to studies employing GenAI and LLMs, such as ChatGPT. The model executes the code within a proprietary environment, limiting the transferability and workflows to other platforms like Google Colab or Jupyter, and may exhibit variability in context retention.

The results of our study suggest that broad systemic parameters are inadequate as direct indicators of localized tissue damage (clinical attachment loss) without the support of localized data. This contrasts with recent advances in deep learning that utilize site-specific imaging data (55, 56). For instance, recent studies analyzing intraoral photographs and panoramic radiographs have achieved significantly higher diagnostic accuracy. These image-based models directly capture local signs of bone loss and inflammation, which are invisible to a model relying solely on systemic metadata.

Consequently, we suggest that the value of system-based GenAI models lies not in diagnosis, but rather in risk stratification. In settings with limited resources where dental radiography is unavailable, our workflow can serve as an initial screening tool to identify patients with elevated systemic risk scores who should receive prioritized clinical periodontal evaluations. Finally, it is important to situate our findings within the broader scientific context. This study represents an *in-silico* analysis based on a retrospective dataset. Accordingly, our results and the predictive models developed are hypothesis-generating and should not be considered clinically reliable without further validation. The performance metrics, although encouraging, reflect statistical associations within this specific dataset and do not ensure real-world clinical applicability. Before clinical implementation, the model would require extensive validation through prospective, multi-center clinical trials to assess its accuracy, safety, and impact on patient outcomes in real-world settings.

## 6 Conclusion

This study examined the use of GenAI to facilitate data preprocessing and the development of machine learning models for predicting periodontitis. Using the retrospective dataset, the generated models achieved moderate predictive accuracy. Subsequent feature importance analysis showed age and blood sugar as the top two predictors, consistent with established risk factors for periodontitis progression.

These findings should be regarded as preliminary. As an *in-silico* analysis of a retrospective dataset, our results are hypothesis-generating, and the models are not clinically validated. Their performance within this dataset does not ensure real-world clinical applicability. This study ultimately redirects attention from the pursuit of a singular 'systemic predictor' to demonstrating a strong, automated workflow driven via GenAI. A replicable pipeline for data processing and modeling has been established. Future investigations should focus on implementing this identical GenAI workflow with multimodal datasets, merging our systemic risk scoring with localized clinical metrics or radiographic imaging. This comprehensive strategy would connect systemic risk factors with local disease presentation, maximizing the capabilities of AI in automated risk screening and clinical data processing.

## Data Availability

The data analyzed in this study is subject to the following licenses/restrictions: The dataset used in this analysis was collected retrospectively from the hospital with ethical approval. The raw data cannot publicly be released due to patient privacy and confidentiality agreements. Subject to a data sharing agreement and ethical approval, the corresponding author may provide access to the data for research purposes upon reasonable request. Access requests should be directed to the corresponding author, Surajit Bhattacharjee, sbhattacharjee@gmail.com.
